# Hepatitis B Hospitalizations in Brazil: Temporal and Regional Patterns from 2008 to 2023

**DOI:** 10.3390/v17030348

**Published:** 2025-02-28

**Authors:** Danielle Satie Kassada, Igor de Lima Peixoto Rocha, Leonardo Dresch Eberhardt

**Affiliations:** School of Nursing, University of Campinas, 126 Tessália Vieira de Camargo Street, Cidade Universitária, Campinas 13083-887, São Paulo, Brazil; i248749@dac.unicamp.br (I.d.L.P.R.); dresch@unicamp.br (L.D.E.)

**Keywords:** hepatitis B, epidemiology, communicable disease control

## Abstract

Hepatitis B remains a significant global public health concern, particularly in low- and middle-income countries, where prevention and control measures often face challenges. In Brazil, substantial efforts have been made over the years to combat the burden caused by hepatitis B through public health interventions, including vaccination programs, antenatal screening, and prevention of vertical transmission. However, despite these advancements, disparities in disease trends persist across regions and vulnerable populations, requiring ongoing analysis and intervention. This study aimed to analyze the trend in hospital admissions for hepatitis B in Brazil from 2008 to 2023. Data were collected from the SUS Hospital Information System. Statistical analyses were conducted using the Joinpoint Regression Program (version 5.0.2), applying a 5% significance level to identify significant trends over the study period. A total of 19,735 hospitalizations for hepatitis B were recorded during the study period. The overall trend showed a significant decline in hospital admissions, reflecting the effectiveness of public health interventions such as expanded vaccination coverage, screening programs, and prevention strategies. Despite this overall decline, notable regional disparities were observed. The midwest region exhibited an increasing trend in hospitalizations, contrasting with the national decline. Furthermore, a concerning rise in hospital admissions among infants under one year of age was identified, indicating potential shortcomings in the prevention of the vertical transmission of the virus. This study highlights both the successes and persistent challenges in controlling hepatitis B hospitalizations in Brazil. Maintaining high vaccination coverage and implementing targeted public health campaigns for vulnerable populations are crucial for sustaining progress. The regional disparities and failures in vertical transmission prevention require continued attention and intervention to advance toward the goal of eliminating hepatitis B as a public health threat in Brazil.

## 1. Introduction

Hepatitis B virus (HBV) infection is primarily transmitted through exposure to infected blood, unprotected sexual contact, unsafe injections, and from mother to child during childbirth. Chronic hepatitis B develops when the immune system fails to clear the virus, leading to persistent liver inflammation. Over time, this can result in cirrhosis, liver failure, or hepatocellular carcinoma. Despite the availability of an effective vaccine, hepatitis B remains a significant public health burden, particularly in low- and middle-income countries where access to vaccination, testing, and treatment is limited [1].

In Brazil, hepatitis B has been a long-standing public health challenge, especially in regions with lower socioeconomic indicators and high rates of health inequity. The disease burden is not evenly distributed across the country, with higher prevalence observed in the northern and central-west regions, where cultural, geographical, and healthcare access factors contribute to increased transmission. These regions often face challenges in implementing prevention strategies, such as vaccination programs, prenatal screening, and harm-reduction initiatives for high-risk populations [2].

Hospital admissions due to hepatitis B reflect the severe progression of the disease, particularly cases involving complications such as cirrhosis or liver cancer [3]. Analyzing the trend in hospitalizations over time can provide insight into the effectiveness of prevention and treatment interventions as well as highlight disparities in healthcare access. In Brazil, efforts to control hepatitis B have included the introduction of universal vaccination for infants in 1998 and expanded vaccination programs targeting at-risk groups, such as healthcare workers and individuals with multiple sexual partners [4].

The integration of hepatitis B testing and treatment into primary healthcare services has played a pivotal role in reducing the burden of disease. However, significant gaps remain, particularly in rural and underserved areas. Many individuals with chronic hepatitis B remain undiagnosed, as the disease often progresses silently without symptoms until complications arise. This delay in diagnosis contributes to increased hospitalizations and mortality. Furthermore, adherence to long-term antiviral treatment, which is critical for managing chronic infection, can be challenging for patients due to financial, logistical, and systemic barriers [5,6].

Globally, the World Health Organization (WHO) has set ambitious targets to eliminate hepatitis B as a public health threat by 2030 [5,6]. These goals include a 90% reduction in new infections and a 65% reduction in hepatitis-related mortality. Achieving these targets requires a multipronged approach, including vaccination, early diagnosis, treatment, and awareness campaigns. In Brazil, aligning with these global strategies will be essential to reduce the burden of hepatitis B and its associated complications, ultimately preventing hospitalizations and saving lives.

The WHO estimates that 254 million people were living with chronic hepatitis B infection in 2022, with 1.2 million new infections each year. In 2022, hepatitis B resulted in around 1.1 million deaths, mainly from cirrhosis and hepatocellular carcinoma (primary liver cancer) [5]. The aim of this study was to analyze the trend in hospital admissions due to hepatitis B in Brazil from 2008 to 2023.

## 2. Methods

### 2.1. Study Design

This study is quantitative, descriptive, and cross-sectional. Data were collected on hepatitis B cases (In Brazil, according to the Ministry of Health, all confirmed cases of hepatitis B, including outbreaks, must be reported within seven days in the Notifiable Diseases Information System (SINAN), using the “Viral Hepatitis Investigation Form”, which must be sent regularly to the local epidemiological surveillance agency. A confirmed case of hepatitis B for notification purposes is an individual who (i) has one or more reactive markers or a molecular biological test (HBV-DNA) for hepatitis B, namely, HBsAg reactive (by rapid test or laboratory test); or reactive anti-HBc IgM; or detectable HBV-DNA; or (ii) died with hepatitis B listed on the death certificate; or (iii) died with hepatitis of unspecified etiology on the death certificate but was confirmed as having hepatitis B after investigation [7]) registered in the Notifiable Diseases Information System across all regions of Brazil from 2008 to 2023 (data extracted on 1 July 2024). Although data were available up to 2023, the notification of new cases significantly decreased due to the COVID-19 pandemic, leading the statistician to exclude 2020 and 2021 from the analysis.

The exclusion of data from 2020 and 2021 reflects the impact of the COVID-19 pandemic on routine health services and disease surveillance systems. During this period, healthcare systems were overwhelmed, leading to disruptions in hepatitis B screening, diagnosis, and reporting. This phenomenon was not unique to Brazil, as similar trends were observed globally, emphasizing the broader implications of the pandemic on communicable disease control. Understanding how these disruptions affected hepatitis B trends is critical to contextualizing the results of this study.

### 2.2. Statistical Analysis

The dataset included key variables such as race/color, age group, and region of notification (geographically, Brazil is divided in five regions: northern, northeast, midwest, southeast, and southern. Northern Brazil includes the states of Acre, Amapá, Amazonas, Pará, Rondônia, Roraima and Tocantins. The Amazon Rainforest, renowned for its biodiversity, covers most of the region; northeast is the Brazilian region with the largest number of states (nine): Alagoas, Bahia, Ceará, Maranhão, Paraíba, Piauí, Pernambuco, Rio Grande do Norte and Sergipe. Due to its different physical characteristics, the region is divided into four sub-regions, Mid-North, Sertão, Agreste and Zona da Mata, with very different levels of human development within its geographical zones. The midwest region consists of three states, Goiás, Mato Grosso and Mato Grosso do Sul, and the Federal District, where Brasília is located. It is the only region that borders all the others, and it is also the most inland region of the country, being the only one without a coastline. The southeast region of Brazil consists of four states, Espírito Santo, Minas Gerais, Rio de Janeiro, and São Paulo, and is the most important industrial, commercial, and financial region in the country. The southern region of Brazil consists of three states, Rio Grande do Sul, Santa Catarina and Paraná, being the only region that is located below the tropical zone, having very delimited seasons, and having social indices above the Brazilian average), which were essential for analyzing hospitalization trends. Joinpoint regression analysis was used to identify significant changes in trends over time, as it allows for detecting points where the direction or magnitude of the trends shift [8]. A general model was created alongside models adjusted for age group, race, and region to provide a detailed understanding of the factors influencing hospitalizations.

Logarithmic transformation of the dependent variable was applied to ensure the assumptions of constant variance and uncorrelated errors were met. The analysis produced the annual percentage change (APC) and the corresponding confidence intervals, which highlight the magnitude and statistical significance of the observed trends. All analyses were performed using Joinpoint Regression Program version 5.0.2, with results considered significant at a 5% significance level.

### 2.3. Ethics Approval

Since the data used were secondary, publicly accessible, non-restricted, and did not contain any identifying information about the participants, this study was exempt from evaluation by the Research Ethics Committee, in accordance with Resolution No. 510 of 7 April 2016, of the National Health Council.

## 3. Results

Between 2008 and 2023, Brazil recorded 19,735 hospitalizations due to hepatitis B. The trend analysis revealed two distinct phases: a pronounced decline in hospitalizations from 2008 to 2012, followed by a more gradual decrease from 2012 to 2023.

Regarding the hospitalization due to hepatitis B in Brazil between 2008 and 2023, there was a downward trend between 2012 and 2023 (APC = −3.99; 95% CI = −5.11; −0.83).

This study’s findings indicate an overall decreasing trend in hepatitis B hospitalizations in Brazil. However, an exception was observed in infants under one year of age and in the central-west region of the country, where a rising trend in hospitalizations was identified (Table 1).

## 4. Discussion

The declining trend in hospitalizations due to hepatitis B in Brazil from 2008 to 2023 serves as a strong indicator of the effectiveness of public health interventions implemented during this period. Brazil has made substantial progress in preventing and managing hepatitis B, largely through the introduction of widespread vaccination programs and blood screening protocols. The observed reduction in hospitalizations likely reflects the success of these initiatives in reducing both the incidence and severity of hepatitis B infections, a trend also noted in countries like China and the United States [9,10,11].

A key strategy has been the inclusion of the hepatitis B vaccine in the National Immunization Program (NIP). The vaccine is administered to newborns, children, and high-risk populations, which has likely contributed significantly to the reduction in severe cases requiring hospitalization. Furthermore, the expansion of antenatal screening and the administration of immunoglobulin to newborns of Hepatitis B-positive mothers have been crucial in preventing mother-to-child transmission, thereby further reducing the disease burden [12].

The analysis showed a significant reduction in hepatitis B hospitalizations among brown people between 2013 to 2023. However, evidence from other studies suggests that whites have higher rates of adherence to hepatitis B vaccination than other populations, highlighting potential disparities in access and adherence to prevention strategies [13,14].

However, this study also revealed significant regional disparities in the trends in hepatitis B hospitalizations, with the central-west region showing a rising trend, contrary to the overall national decline. This phenomenon has been observed in other studies as well [15,16], highlighting the need for a closer examination of the underlying causes and the formulation of targeted public health interventions.

Several factors may contribute to the rising trend in the central-west region. This area has experienced rapid population growth and urbanization, potentially straining healthcare infrastructure and resources. Additionally, disparities in healthcare access and quality across different regions could have led to differences in the detection and management of hepatitis B cases. The central-west region may also have unique epidemiological characteristics, such as a larger indigenous population with historically lower vaccination coverage, which could have contributed to higher transmission rates.

The growing trend in hospitalizations in this region underscores the need for region-specific strategies to combat hepatitis B. These strategies could involve enhancing vaccination efforts, particularly in underserved communities, improving access to healthcare services, and conducting targeted public health campaigns to raise awareness about hepatitis B prevention and treatment [16].

Studies indicate that indigenous populations in the Americas have a higher prevalence of the HBV F genotype, which is often associated with the adw4 subtype of HBsAg. The hepatitis B vaccine, which is based on the A2 genotype and the adw2 subtype, may have reduced immunogenicity against divergent genotypes such as F, potentially contributing to vaccine breakthrough infections. Evidence suggests that this antigenic difference may be associated with an increased risk of vertical transmission, particularly in contexts of low vaccination coverage with doses at birth and the lack of hepatitis B immunoglobulin (HBIG) administration. These findings underscore the need for enhanced surveillance and immunization strategies adapted to vulnerable populations [17].

Moreover, this study identified an increasing trend in hospitalizations among infants under one year of age, a finding that is particularly concerning and underscores the need for focused attention on this vulnerable population. Although overall hepatitis B hospitalizations are declining, the rising trend in this age group suggests potential gaps in the prevention of mother-to-child transmission (MTCT) of hepatitis B, a concern also noted in studies conducted in China [4,9]. In another study, the global estimate of hepatitis B prevalence showed a significant reduction, especially among children under 5 years of age, according to data from the Global Burden of Disease [18].

While Brazil has implemented comprehensive strategies to prevent MTCT, including antenatal screening and the administration of the hepatitis B vaccine and immunoglobulin to newborns, the increasing hospitalizations in infants suggest that these measures may not be reaching all at-risk populations. Barriers such as limited access to antenatal care, particularly in remote or underserved areas, could result in missed opportunities for intervention. It is also important to note that Brazil has experienced a reduction in vaccination coverage, significantly impacted during the COVID-19 pandemic, and efforts are ongoing to restore the high coverage levels achieved in the past.

This reduction has been mainly attributed to the expansion of hepatitis B vaccination coverage in several countries [19], which has been an important milestone in preventing the vertical and horizontal transmission of the virus in this age group. However, the results of this study show a different trend, with prevalence patterns that do not follow the same downward trajectory observed worldwide. This discrepancy may be related to regional factors, such as inequities in access to vaccination, challenges in implementing public health policies, or limitations in early diagnosis and surveillance of the disease. Therefore, these results highlight the importance of studying contextual factors and evaluating the effectiveness of local strategies to control hepatitis B, especially in vulnerable populations.

Addressing this issue requires strengthening maternal and child health services, ensuring that all pregnant women are screened for hepatitis B and that newborns receive timely prophylaxis. Additionally, there is a need for continuous monitoring and evaluation of the effectiveness of the existing MTCT prevention programs to identify and address gaps. A study conducted in Brazil with pregnant women identified that vaccination coverage is influenced not only by individual factors but also by geographical barriers and the distance between the home and healthcare facilities, which can contribute to vaccine hesitancy [2].

An analysis of hospital admissions for hepatitis B among the elderly in Brazil showed a downward trend in recent years, indicating progress in the control and management of the disease in this age group [20]. This decline may be related to the positive impact of public policies, such as the extension of hepatitis B vaccination to vulnerable populations and the increased provision of early diagnosis and appropriate treatment in primary health care. In addition, awareness of the modes of transmission and the implementation of educational strategies may have contributed to the reduction in new cases and complications in older adults. These findings underscore the importance of integrated and sustained interventions to control infectious diseases, especially in more vulnerable populations.

The findings of this study have significant implications for public health policy and practice in Brazil. While the overall decline in hepatitis B hospitalizations suggests that current prevention and management strategies are effective, the identified disparities indicate that these efforts need to be tailored to address specific regional and demographic challenges.

To sustain and further enhance the progress made in controlling hepatitis B, it is essential to maintain high vaccination coverage, particularly in high-risk populations and regions with rising trends. Public health campaigns should be intensified in regions like the central-west, where the burden of disease appears to be increasing. Additionally, the healthcare system must ensure that maternal and child health services are accessible to all, particularly in remote and underserved areas, to prevent MTCT and protect infants from hepatitis B.

This study has some limitations. First, the use of secondary data from the SUS Hospital Information System may be subject to under-reporting and misclassification. Second, hospital admission data do not capture asymptomatic or mild cases managed in outpatient settings, potentially underestimating the disease burden. Third, regional disparities in healthcare access and reporting practices may influence trend analysis. Fourth, the joinpoint regression analysis is limited by data quality and potential changes in coding practices over time. Fifth, this study did not assess individual patient characteristics, which could provide deeper insights into risk factors. Finally, the findings may not fully represent the private healthcare sector, limiting generalizability.

Future research should explore the data on prevalence and incidence of hepatitis B, as well as the factors driving the regional and age-specific trends observed in this study, including sociodemographic, economic, and healthcare-related determinants. Understanding these factors will be critical in developing targeted interventions to reduce the burden of hepatitis B across all regions of Brazil.

## 5. Conclusions

In conclusion, while the overall trend of declining hepatitis B hospitalizations in Brazil is encouraging, this study highlights the need for continued vigilance and targeted public health efforts to address persistent and emerging challenges in specific regions and populations. By addressing these challenges, Brazil can make significant strides toward the goal of eliminating hepatitis B as a public health threat. The central-west region, in particular, demands immediate attention due to its rising hospitalization rates. Public health authorities must allocate additional resources to this region to bolster vaccination programs, improve healthcare access, and enhance surveillance systems that can quickly identify and address emerging outbreaks.

Efforts to prevent mother-to-child transmission must also be reinforced to protect infants, as rising hospitalization rates in this vulnerable group suggest critical gaps in the healthcare delivery system. Policymakers should prioritize strengthening antenatal care services and ensuring that newborns receive timely immunization and prophylaxis. Moreover, addressing broader determinants such as socioeconomic disparities, healthcare infrastructure limitations, and geographic barriers will be essential to achieving equitable health outcomes across all regions. Brazil’s success in controlling hepatitis B depends on a comprehensive, evidence-based approach that integrates prevention, monitoring, and treatment strategies to reach all populations, particularly those most at risk.

## Figures and Tables

**Table 1:** Cases of hospital admissions due to hepatitis B by race/ethnicity, age group, and region in Brazil, 2008–2023.

Color/Race	Tendency ^a^	Period	APC ^b^ (%)	Confidence Interval (95%)	
				Lower Limit	Upper Limit
Caucasian	1	2008–2011	−34.81	−50.02	−21.72
	2	2011–2023	−1.73	−4.27	1.62
Black	1	2008–2010	18.07	−3.85	44.76
	2	2010–2013	−25.09	−30.33	−13.21
	3	2013–2023	0.28	−2.54	4.70
	1	2008–2010	−0.68	−11.69	2.98
Brown	2	2010–2013	14.28	5.57	21.14
	3	2013–2023	−4.57	−9.78	−1.67
Yellow	1	2008–2014	−19.32	−60.03	1.40
	2	2014–2023	14.28	−1.58	57.61
Indigenous	1	2008–2023	−8.16	−17.41	1.26
**Age Group**	**Tendency ^a^**	**Period**	**APC ^b^ (%)**	**Confidence Interval (95%)**	
				**Lower Limit**	**Upper Limit**
<1	1	2008–2019	−4.90	−13.05	−0.54
	2	2019–2023	40.76	12.91	59.33
1 to 9	1	2008–2018	−13.71	−32.14	−9.26
	2	2018–2023	6.50	−7.58	24.90
10 to 19	1	2008–2023	−8.21	−11.02	−5.43
20 to 59	1	2008–2012	−17.68	−29.28	−11.01
	2	2012–2023	−4.44	−6.27	−1.47
60 or more	1	2008–2012	−20.01	−34.47	−12.39
	2	2012–2018	2.69	−1.00	17.82
	3	2018–2023	−6.30	−13.00	−2.13
**Region**	**Tendency ^a^**	**Period**	**APC ^b^ (%)**	**Confidence Interval (95%)**	
				**Lower Limit**	**Upper Limit**
North	1	2008–2014	3.40	−0.97	10.89
	2	2014–2017	−32.64	−37.81	−21.65
	3	2017–2023	1.39	−3.57	10.80
Northeast	1	2008–2013	−17.87	−23.52	−14.11
	2	2013–2018	5.99	1.69	17.15
	3	2018–2023	−13.93	−17.92	−10.48
Southeast	1	2008–2010	−39.71	−47.20	−21.78
	2	2010–2023	−1.70	−3.77	1.04
South	1	2008–2010	−40.93	−50.81	−15.63
	2	2010–2023	−8.41	−10.98	−3.02
Midwest	1	2008–2019	−5.57	−26.09	0.31
	2	2019–2023	27.83	1.16	46.31

Source: DATASUS. Notifiable Diseases Information System of the Brazilian Unified Health System (SUS) from 2008 to 2023. Legend: ^a^. period of time during which the trend followed a certain pattern; ^b^. annual percentage change.

## Data Availability

The database can be found in the Datasus Tabnet system at the following website: https://datasus.saude.gov.br/informacoes-de-saude-tabnet/ (accessed on 7 February 2025).
